# Plastid Anionic Lipids Are Essential for the Development of Both Photosynthetic and Non-Photosynthetic Organs in *Arabidopsis thaliana*

**DOI:** 10.3390/ijms22094860

**Published:** 2021-05-04

**Authors:** Akiko Yoshihara, Noriko Nagata, Hajime Wada, Koichi Kobayashi

**Affiliations:** 1 Department of Biological Science, Graduate School of Science, Osaka Prefecture University, 1-1 Gakuencho, Naka-ku, Sakai 599-8531, Japan; sbc04113@edu.osakafu-u.ac.jp; 2 Department of Chemical and Biological Sciences, Faculty of Science, Japan Women’s University, 2-8-1 Mejirodai, Bunkyo-ku, Tokyo 112-8681, Japan; n-nagata@fc.jwu.ac.jp; 3 Department of Life Sciences, Graduate School of Arts and Sciences, The University of Tokyo, 3-8-1 Komaba, Meguro-ku, Tokyo 153-8902, Japan; hwada@g.ecc.u-tokyo.ac.jp; 4 Faculty of Liberal Arts and Sciences, Osaka Prefecture University, 1-1 Gakuen-cho, Naka-ku, Sakai, Osaka 599-8531, Japan

**Keywords:** arabidopsis, chloroplast, lipid, phosphorus starvation, phosphatidylglycerol, photosynthesis, plastid, sulfoquinovosyldiacylglycerol, thylakoid membrane

## Abstract

The lipid bilayer matrix of the thylakoid membrane of cyanobacteria and chloroplasts of plants and algae is mainly composed of uncharged galactolipids, but also contains anionic lipids sulfoquinovosyldiacylglycerol (SQDG) and phosphatidylglycerol (PG) as major constituents. The necessity of PG for photosynthesis is evident in all photosynthetic organisms examined to date, whereas the requirement of SQDG varies with species. In plants, although PG and SQDG are also found in non-photosynthetic plastids, their importance for the growth and functions of non-photosynthetic organs remains unclear. In addition, plants synthesize another anionic lipid glucuronosyldiacylglycerol (GlcADG) during phosphorus starvation, but its role in plant cells is not elucidated yet. To understand the functional relationships among PG, SQDG, and GlcADG, we characterized several *Arabidopsis thaliana* mutants defective in biosynthesis of these lipids. The mutants completely lacking both PG and SQDG biosynthesis in plastids showed developmental defects of roots, hypocotyls, and embryos in addition to leaves, which suggests that these lipids are pleiotropically required for the development of both photosynthetic and non-photosynthetic organs. Furthermore, our analysis revealed that SQDG, but not GlcADG, is essential for complementing the role of PG, particularly in photosynthesis under PG-deficient conditions such as phosphorus starvation.

## 1. Introduction

Thylakoid membranes of both cyanobacteria and plant chloroplasts contain four major lipids, monogalactosyldiacylglycerol (MGDG), digalactosyldiacylglycerol (DGDG), sulfoquinovosyldiacylglycerol (SQDG), and phosphatidylglycerol (PG). Uncharged galactolipids (MGDG and DGDG) consist ~80 mol% of total thylakoid glycerolipids, and SQDG and PG, which are anionic lipids with a negative charge in polar head groups, account for the rest ~20 mol% [1]. In plants, a few percent of phosphatidylinositol is also found in the thylakoid membrane [2].

PG is essential for photosynthesis and growth of all oxygenic phototrophs examined to date [3]. In both cyanobacteria and plants, phosphatidylglycerophosphate (PGP) synthase catalyzes the conversion of cytidine diphosphate-diacylglycerol (CDP-DAG) and glycerol-3-phosphate to PGP, which is subsequently dephosphorylated to PG by PGP phosphatase. Genes for PGP synthase have been well characterized particularly in model organisms *Arabidopsis thaliana* and *Synechocystis* sp. PCC 6803 [3], although genes responsible for the PGP phosphatase activity are still not fully revealed in both plants and cyanobacteria [4,5,6]. Knockout mutations of the PGP synthase gene (*pgsA*) in cyanobacteria severely impaired formation and functions of photosystems, particularly photosystem II (PSII) and cell growth [7,8,9]. In *A. thaliana*, two homologs for PGP synthase have been identified and designated as PGP1 and PGP2 [10]. PGP1 is dually targeted to plastids and mitochondria [11], whereas PGP2 is targeted to the endoplasmic reticulum [12]. A single amino acid substitution (P170S) of PGP1 by a point mutation (*pgp1-1*) reduces its activity by 80%, which causes a 30% reduction of overall PG content and a slight decrease in photosynthetic activity in *A. thaliana* [13]. A knockout mutation of the *PGP1* gene by a T-DNA insertion (*pgp1-2*) decreased PG content in leaves by 88% of the wild-type level, which resulted in severe impairments of thylakoid membrane development, chlorophyll accumulation, photochemical reactions of PSII, and the photosynthetic electron transport [14,15,16]. The *pgp1-2* mutant absolutely requires supplementation of sugar for growth, and even in the presence of sugar, its growth is stunted at seedling stages, resulting in seedling lethality. In contrast to severely disrupted chloroplasts, mitochondria are functional in *PGP1* knockout mutants [11,14]. Because mitochondria of the *PGP1* knockout mutant accumulated cardiolipin, the mitochondrion-specific lipid synthesized from PG, to the wild-type level [11], ER-localized PGP2 would function in PG biosynthesis to support the activity of mitochondria. In fact, although the single *pgp2* knockout mutant showed no obvious defective phenotype, the *pgp1-2 pgp2* double mutant could not produce PG and cardiolipin and caused embryonic lethality [12]. These data suggest a subsidiary role of the ER pathway by PGP2 in *A. thaliana*.

In contrast to the necessity of PG in plants and cyanobacteria, the requirement of SQDG for photosynthesis and growth varies with species [1]. SQDG is synthesized in two steps: first, sulfite and the uridine diphosphate (UDP)-glucose are converted into UDP-sulfoquinovose (UDP-SQ), and next, the SQ moiety of UDP-SQ is transferred to DAG to yield SQDG. In *A. thaliana*, the first and second reactions are catalyzed by SQD1 and SQD2, respectively, each of which is encoded by a single gene. The knockout mutants of *SQD1* and *SQD2* both completely lacked SQDG but showed wild type-like phenotypes under nutrient-sufficient conditions [17,18], indicating that SQDG is not essential for growth of *A. thaliana* under optimal conditions. In these mutants, loss of SQDG was accompanied by increased PG content. The complementary increase in PG in response to loss of SQDG was also observed in cyanobacteria [9,19,20] and green algae *Chlamydomonas reinhardtii* [21,22], which implies that PG substitutes for the decreased SQDG, as they share common features of anionic lipids. Consistent with these observations, when the *pgp1-1* mutation was introduced into the *sqd2* mutant, the resulting *sqd2 pgp1-1* double mutant showed stronger impairments in photosynthesis and growth than each single mutant with decreased total anionic lipid content [23]. The data further confirm a complementary relationship between SQDG and PG and suggest an importance of maintaining total amount of anionic lipids in membranes.

Under phosphorus (P)-starved conditions, the PG content decreases and conversely the SQDG content increases in various photosynthetic organisms, including *A. thaliana*. In *C. reinhardtii* [22] and cyanobacteria [9,20], a lack of SQDG biosynthesis by genetic disruptions enhances the growth impairment under P-starved conditions, suggesting that the compensation for decreased PG by increased SQDG is important for the growth under P starvation. The *sqd2* mutants of *A. thaliana* also showed stronger growth retardation than wild type under P starvation [17,18]. However, the growth of the *sqd1* mutant under P starvation was similar to that of wild type [18], although this mutant lacks SQDG as does *sqd2*. Okazaki et al. (2013) revealed that *A. thaliana* wild type and *sqd1*, but not *sqd2*, accumulate another anionic lipid glucuronosyldiacylglycerol (GlcADG) under P-starved conditions [18]. From these findings, the authors proposed that SQD2 catalyze the biosynthesis of GlcADG in addition to SQDG, presumably by using DAG and UDP-glucuronate as substrates. They speculated that GlcADG has an important role in plant growth under P-starved conditions, and, thus, the lack of GlcADG causes growth impairment in P-starved *sqd2*.

The much stronger growth impairment of the *sqd2 pgp1-1* double mutant than each single mutant suggests an importance of compensation between SQDG and PG in *A. thaliana* [23]. However, SQD2 has been implicated to be responsible for the biosynthesis of GlcADG in addition to SQDG, and what extent GlcADG can contribute to the complementary relationship between SQDG and PG was unknown. Moreover, because *pgp1-1* is a leaky mutant having 30% PGP synthesizing activity compared with wild type [13], the *sqd2 pgp1-1* mutant still retains the ability to synthesize PG in plastids. Therefore, the effects of the lack of SQDG with complete loss of plastid PG biosynthesis on plant growth remain undetermined. To understand the functions of anionic lipids synthesized in plastids in plant growth, we generated double mutants of *sqd1 pgp1-1*, *sqd2 pgp1-1*, *sqd1 pgp1-2*, and *sqd2 pgp1-2* (Table 1) and compared the phenotypes of these mutants, including those in the development of non-photosynthetic organs.

## 2. Results

### 2.1. Loss of SQD1 and SQD2 in the pgp1-1 Background Causes the Same Growth Defects under P-sufficient Conditions

**Figure 1:**
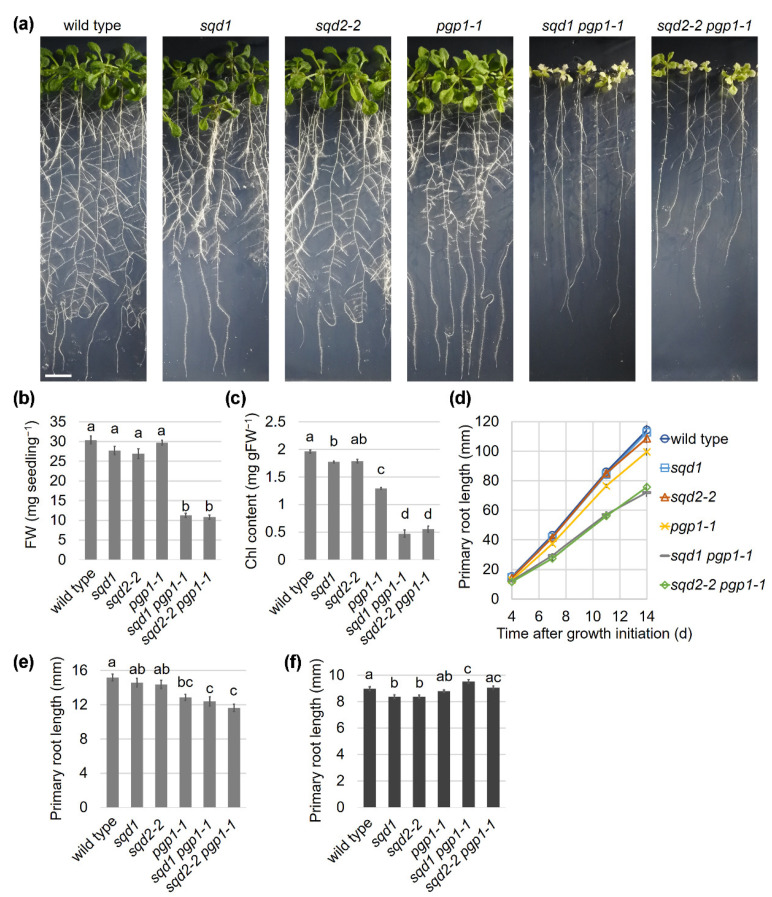
Comparison of growth characteristics of wild type and anionic lipid mutants. (**a**) Visible phenotypes of 14-day-old seedlings. All photographs are in the same scale (bar = 10 mm). (**b**) Fresh weight (FW) and (**c**) chlorophyll (Chl) content of the shoot of 14-day-old seedlings. Data are means ± SE of 24 independent samples. (**d**) Changes of primary root length during seedling growth under light. (**e**) Primary root length of 4-d-old seedlings grown under light. In (**d**) and (**e**), data are means ± SE of 36 seedlings. (**f**) Primary root length of 4-d-old seedlings grown under the dark condition. Data are means ± SE of 299, 290, 337, 359, 321, and 298 seedlings for wild type, *sqd1*, *sqd2-2*, *pgp1-1*, *sqd1 pgp1-1*, and *sqd2-2 pgp1-1*, respectively. In (**b**,**c**,**e**,**f**), the lowercase letters indicate statistically significant differences between each line (*p* < 0.05, Tukey’s post hoc honestly significant difference test).

Although Yu and Benning (2003) reported that the *sqd2 pgp1-1* double mutant showed more severe growth defects than each single mutant [23], to what extent the loss of GlcADG biosynthesis contributed to the defective phenotype of the double mutant was unknown. To assess whether GlcADG has any functions under P-sufficient conditions, we compared growth phenotypes of 14-d-old seedlings of *pgp1-1*, *sqd1*, *sqd2-2*, and their double mutants with wild type. Visible phenotypes of *sqd1* and *sqd2-2*, which carry a T-DNA insertion at the first exon of *SQD1* and *SQD2*, respectively [18,24], were almost comparable to that of wild type (Figure 1a). Fresh weight (FW) of the above-ground part of *sqd1* and *sqd2-2* appeared slightly lower than that of wild type, but with a significant difference only between *sqd2-2* and wild type (Figure 1b). Chlorophyll (Chl) content in *sqd1* and *sqd2-2* was slightly but significantly lower than that in wild type (Figure 1c). Measurements of primary root length indicate no difference among wild type, *sqd1*, and *sqd2-2* in root growth (Figure 1d). The *pgp1-1* mutant showed a ~30% reduction in Chl content compared to wild type (Figure 1c), although its FW was not significantly different from the wild-type level (Figure 1b). Elongation of the primary root slightly decreased in *pgp1-1* compared with wild type (Figure 1d). The double homozygous mutants of *sqd1 pgp1-1* and *sqd2-2 pgp1-1*, which were generated by crossing *sqd1* or *sqd2-2* with *pgp1-1*, showed a pale yellow-green leaf phenotype (Figure 1a), with their FW and Chl content reduced by ~70% and ~75%, respectively, from the wild-type level (Figure 1b,c). Primary root growth of *sqd1 pgp1-1* and *sqd2-2 pgp1-1* further decreased from the *pgp1-1* single mutant (Figure 1d). Meanwhile, no significant differences were observed between *sqd1 pgp1-1* and *sqd2-2 pgp1-1* in FW, Chl content, and primary root length.

The *pgp1-1*, *sqd1 pgp1-1*, and *sqd2-2 pgp1-1* mutants showed shorter primary root length than wild type already at 4 d after growth initiation (Figure 1e). To eliminate the effect of photosynthesis on root growth, we grew wild type and the mutants in complete darkness for 4 d. In the dark condition, both *sqd1* and *sqd2-2* showed a statistically significant reduction of the primary root length compared with wild type, although the difference was marginal (~95% of the wild-type level) (Figure 1f). In contrast, *pgp1-1*, *sqd1 pgp1-1*, and *sqd2-2 pgp1-1* mutants showed no reduced elongation of the primary root compared with wild type. Thus, the reduced primary root growth of these mutants was dependent on light.

### 2.2. Loss of SQD1 and SQD2 Causes Similar Photosynthetic Defects

**Figure 2:**
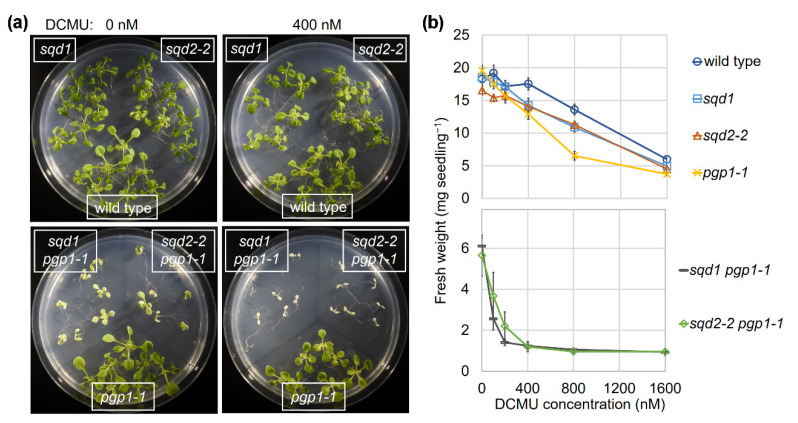
Growth impairment in wild type and anionic lipid mutants by a photosynthesis electron transport inhibitor 3(3,4-dichlorophenyl)-1,1-dimethylurea (DCMU). Seven-d-old seedlings were transferred to DCMU-containing media and grown for another 7 d. (**a**) Growth phenotype of each line in the absence (0 nM) and presence (400 nM) of DCMU. (**b**) Fresh weight of each line under different DCMU concentrations. Data are means ± SE of 14 seedlings for each line from two independent experiments.

Yu and Benning (2003) reported that the *pgp1-1* and *sqd2* mutants were slightly more susceptible to 3(3,4-dichlorophenyl)-1,1-dimethylurea (DCMU), a photosynthesis electron transport inhibitor, than wild type in terms of growth impairment [23]. Moreover, the growth of the *sqd2 pgp1-1* double mutant was severely impaired by DCMU. To assess whether the lack of GlcADG biosynthesis in *sqd2* mutants is associated with the growth impairment by DCMU, we analyzed the effects of several concentrations of DCMU on the growth of wild type and the mutants (Figure 2). The *sqd1* and *sqd2-2* mutants showed a stronger growth impairment by DCMU than wild type, and the growth impairment of *pgp1-1* was more severe than *sqd1* and *sqd2-2*. Moreover, both *sqd1 pgp1-1* and *sqd2-2 pgp1-1* showed an intensive growth impairment even under low DCMU concentrations.

**Figure 3:**
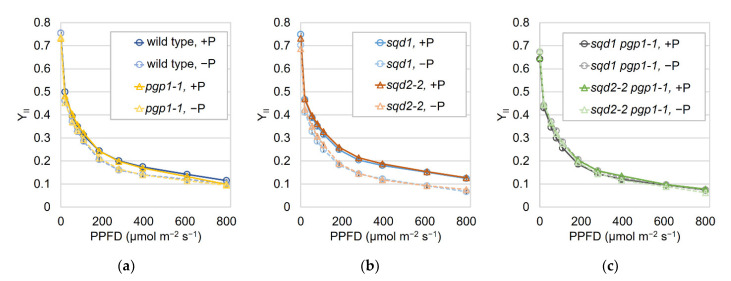
Light–response curves of effective photosystem II quantum yield (Y_II_) in wild type and anionic lipid mutants grown under P-sufficient (+P) and -starved (−P) conditions. (**a**,**b**) Wild type, *pgp1-1*, *sqd1*, and *sqd2-2* were grown for 7 d and (**c**) *sqd1 pgp1-1* and *sqd2-2 pgp1-1* were grown for 11 d under the +P condition, followed by the growth under the +P or −P condition for 7 d. Data are means ± SE of 13, 15, 21, 19, 20, and 22 seedlings for the +P condition and 6, 8, 20, 17, 22, and 26 seedlings for the −P condition of wild type, *pgp1-1*, *sqd1*, *sqd2-2*, *sqd1 pgp1-1*, and *sqd2-2 pgp1-1*, respectively. PPFD, photosynthetic photon flux density.

To further dissect the effect of loss of SQDG and GlcADG biosynthesis on photosynthesis, we grew plants under P-sufficient and -starved conditions and analyzed the light–response curves of effective quantum yield of PSII (Y_II_) with the imaging pulse amplitude modulation Chl fluorometer (imaging-PAM) (Figure 3). In wild type, the Y_II_ levels were slightly decreased by P starvation compared with those under the P-sufficient condition. The light–response curves of Y_II_ in *pgp1-1* were almost the same as those in wild type under each P condition. The *sqd1* and *sqd2-2* mutants also showed the Y_II_ curves similar to those in wild type and *pgp1-1* under the P-sufficient condition. However, under the P-starved condition, the Y_II_ levels in *sqd1* and *sqd2-2* decreased more strongly than wild type and *pgp1-1*. Between *sqd1* and *sqd2-2*, no remarkable difference was observed under both P conditions. In the *sqd1 pgp1-1* and *sqd2-2 pgp1-1* double mutants, the Y_II_ levels were lower than those in wild type and single mutants under the P-sufficient condition. In these double mutants, no further decrease in Y_II_ was observed in response to P starvation. In addition, no noticeable difference was observed between *sqd1 pgp1-1* and *sqd2-2 pgp1-1*.

### 2.3. Complete Loss of Anionic Lipid Biosynthesis in Plastids Substantially Impairs Plant Development

The comparisons of *sqd1 pgp1-1* and *sqd2-2 pgp1-1* revealed that SQDG, but not GlcADG, plays a pivotal role in compensating the decrease in plastid PG biosynthesis in the *pgp1-1* mutant. However, *pgp1-1* retains 30% activity of PGP1; thus, the effect of complete lack of anionic lipid biosynthesis in plastids was undetermined. To further understand the roles of plastid anionic lipids in plant growth, we introduced the knockout *pgp1-2* mutation into *sqd1* and *sqd2-2* and characterized the resulting double mutants.

**Figure 4:**
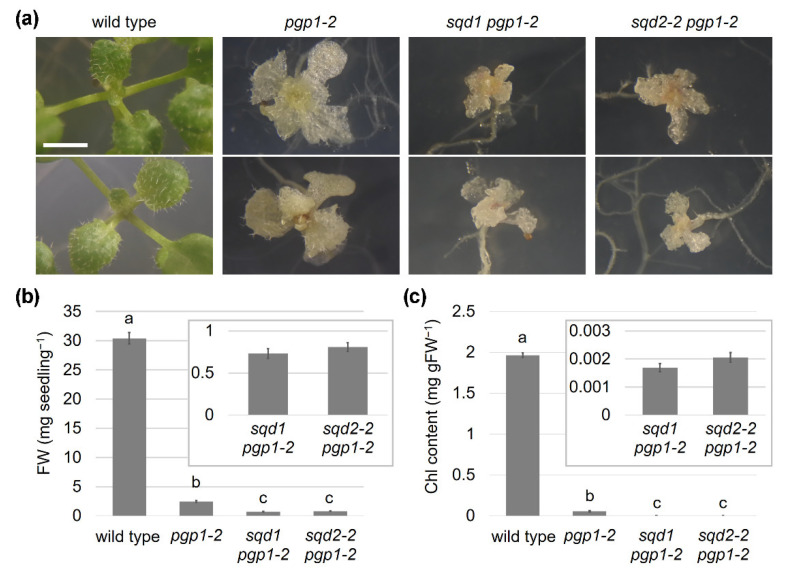
Growth defects of knockout mutants for anionic lipid biosynthesis in plastids. (**a**) Visible phenotypes of 24-d-old seedlings of mutants. Seedlings of 14-d-old wild type were shown for comparison. Two representative individuals were displayed for each line. Scale bar = 2 mm. (**b**) Fresh weight (FW) and (**c**) chlorophyll (Chl) content of the shoot of 14-d-old seedlings. Data are means ± SE of 24 independent samples. Insets focus the data for the *sqd1 pgp1-2* and *sqd2-2 pgp1-2* mutants. The data of wild type in Figure 1 was shown for comparison.

Because the homozygous *pgp1-2* mutant is seedling lethal, this allele is maintained in the heterozygous state. Consistent with previous reports [14,15,16], the homozygous *pgp1-2* mutants, which were obtained from self-pollinated heterozygous parents, showed a severe impairment of growth and Chl accumulation even in the presence of 1% sucrose in the growth medium (Figure 4a–c).

By crossing the heterozygous *pgp1-2* plants with the homozygous *sqd1* and *sqd2-2* mutants and subsequent segregation, we obtained heterozygous *pgp1-2* plants with homozygous *sqd1* or *sqd2-2* mutation (*sqd1*/*sqd1 PGP1/pgp1-2* and *sqd2-2*/*sqd2-2 PGP1/pgp1-2*). In the progeny of these plants, we identified double homozygous mutants of *sqd1 pgp1-2* (*sqd1*/*sqd1 pgp1-2*/*pgp1-2*) and *sqd2-2 pgp1-2* (*sqd2-2*/*sqd2-2 pgp1-2*/*pgp1-2*), both of which showed a severe growth impairment and an albino phenotype (Figure 4a). The *pgp1-2* single mutant developed only few small leaves even at 24 d after growth initiation and the *sqd1 pgp1-2* and *sqd2-2 pgp1-2* double mutants showed stronger growth defects than *pgp1-2*. The leaves of both double mutants were severely underdeveloped with irregular shapes and rough outlines. FW of these double mutants was substantially low and Chl content was almost negligible (Figure 4b,c).

### 2.4. The Photosystem II Function Is Completely Abolished by Loss of Anionic Lipid Biosynthesis in Plastids

**Figure 5:**
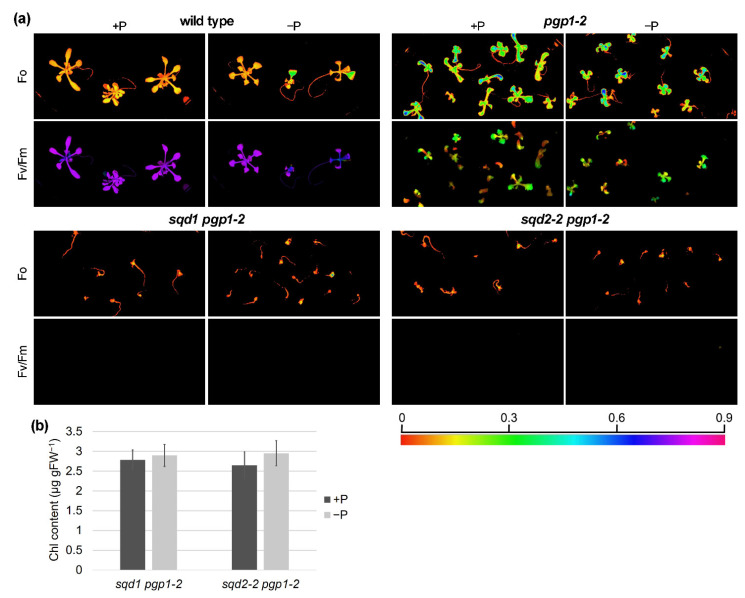
Photosynthetic activity and chlorophyll (Chl) accumulation in the knockout mutants of anionic lipid biosynthesis in plastids. Wild-type seedlings were grown for 7 d and *pgp1-2*, *sqd1 pgp1-2*, and *sqd2-2 pgp1-2* mutants were grown for 14 d under the P-sufficient (+P) condition, followed by the growth under the +P or the P-starved (−P) condition for 7 d. (**a**) Minimum Chl fluorescence (Fo) and maximum PSII quantum yield (Fv/Fm) in seedlings grown under +P or −P conditions. No Fv/Fm signal was observed in the *sqd1 pgp1-2* and *sqd2-2 pgp1-2* seedlings. (**b**) Chl content in the *sqd1 pgp1-2* and *sqd2-2 pgp1-2* seedlings grown under +P or −P conditions. Data are means ± SE of 5 seedlings for each experiment. No statistical difference was observed (*p* < 0.05, Tukey’s post hoc honestly significant difference test).

We previously reported that loss of plastid PG biosynthesis causes a severe but not complete dysfunction of PSII photochemistry [15,16], as shown in a decrease in the maximum PSII quantum efficiency (Fv/Fm) in this mutant under the P-sufficient condition (Figure 5a). As observed previously [15], the *pgp1-2* mutant showed high minimum Chl fluorescence (Fo) because of a severe inhibition of energy transfer from the antenna system to the reaction center in photosystems and consequent dissipation of absorbed light energy as fluorescence. Under the P-starved condition, some *pgp1-2* plants showed complete loss of the PSII photochemical activity, although there were large individual differences. In both *sqd1 pgp1-2* and *sqd2-2 pgp1-2* double mutants, no PSII activity was observed regardless of P conditions, suggesting that SQDG is essential for the remaining PSII activity in the *pgp1-2* mutant.

Our previous study revealed that P starvation activates biosynthesis of glycolipids including SQDG and enhances thylakoid formation and Chl accumulation in *pgp1-2* leaves [16]. To ascertain whether SQDG is required for the activation of chloroplast development in *pgp1-2* under P starvation, we analyzed Chl content in the *sqd1 pgp1-2* and *sqd2-2 pgp1-2* mutants under P-sufficient and -starved conditions (Figure 5b,c). No difference in Chl content between the two P conditions in these mutants demonstrated that SQDG, but not GlcADG, is important for the complementation of decreased PG content under P-starved conditions.

### 2.5. Anionic Lipids Are Essential for Thylakoid Membrane Biogenesis

**Figure 6:**
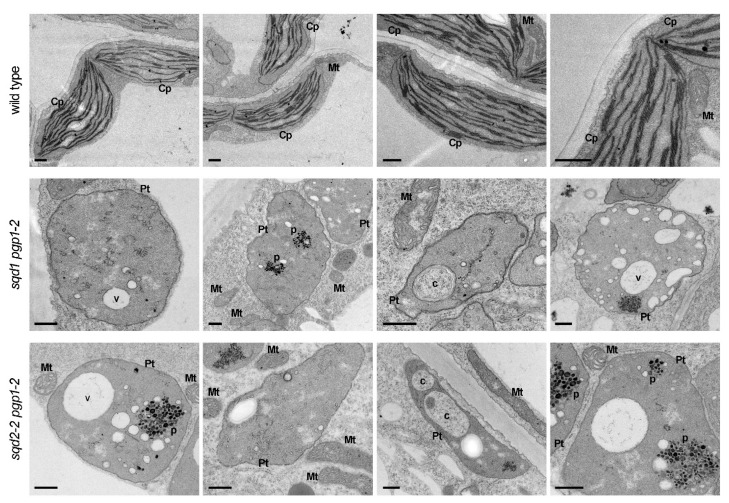
Ultrastructures of plastids and mitochondria in leaves of *sqd1 pgp1-2* and *sqd2-2 pgp1-2* mutants grown for 21 d. The structure of these organelles in wild-type leaves was shown for reference. Cp, chloroplast; Pt, plastid; Mt, mitochondrion; c, cytosol-like structure; p, plastoglobule; v, vacuole-like structure. Scale bars = 500 nm.

We previously reported that plastids in the *pgp1-2* leaves are unable to form thylakoid membrane networks and only partially develop immature internal membrane structures [14,16]. By contrast, the morphology of mitochondria appeared normal in *PGP1* knockout mutants [11,14]. To understand how the lack of SQDG in addition to PG affects the morphology of plastids and mitochondria in leaf cells, we examined the ultrastructure of these organelles in leaves of *sqd1 pgp1-2* and *sqd2-2 pgp1-2* by transmission electron microscopy. In both mutants, leaf plastids developed no thylakoid membranes and instead accumulated many small membrane fragments (Figure 6). Some plastids showed clusters of high electron-dense particles, which resembled plastoglobuli. In the leaf plastids of these double mutants, vacuole-like large electron transparent areas were often observed in addition to vesicle-like small structures. Some plastids also showed cytosol-like inclusions inside. These structures may reflect intrusions of membranous tubes of cytosol or vacuole compartment into plastids, which were not observed in wild-type chloroplasts. In contrast to the abnormal plastid structures, the morphology of mitochondria in these double mutants was similar to that in wild type.

### 2.6. Root Growth Is Impaired in Anionic Lipid Mutants

**Figure 7:**
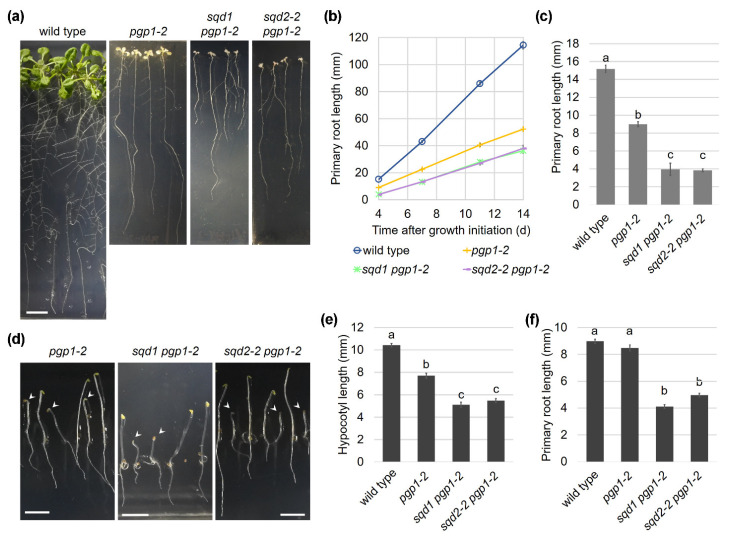
Impaired root growth of knockout mutants of anionic lipid biosynthesis in plastids. (**a**) Root phenotype of each line grown under light for 14 d. All photographs are in the same scale (bar = 10 mm). (**b**) Changes in primary root length during seedling growth under light. (**c**) Primary root length of 4-d-old seedlings grown under light. In (**b**,**c**), data are means ± SE of 194, 80, and 84 seedlings for *pgp1-2*, *sqd1 pgp1-2*, and *sqd2-2 pgp1-2*, respectively. (**d**) Phenotype of etiolated seedlings of each line grown in the dark for 4 d. Arrowheads indicate seedlings with the homozygous *pgp1-2* allele for each line. Scale bar = 5 mm. (**e**,**f**) length of primary root (**e**) and hypocotyl (**f**) of 4-d-old seedlings grown under dark. Data are means ± SE of 299, 205, 81, and 112 seedlings for wild type, *pgp1-2*, *sqd1 pgp1-2*, and *sqd2-2 pgp1-2*, respectively. In (**b,c,f**), the data for wild type in Figure 1 were shown for reference.

In addition to the shoot growth, the primary root growth was severely impaired in *pgp1-2*, and this phenotype was further enhanced in the *sqd1 pgp1-2* and *sqd2-2 pgp1-2* double mutants (Figure 7a,b). In these mutants, root elongation was strongly impaired from the early stage of growth under light (Figure 7c). To eliminate the effect of light on plant growth, we grew these mutants in complete darkness for 4 d and analyzed the phenotypes. The development of the mutants was impaired even in the dark (Figure 7d). The hypocotyl length of the *pgp1-2* mutant was 75% of the wild-type level and those of the double knockout mutants further reduced by half (Figure 7e). Moreover, the double knockout mutants also showed ~50% reduction in root length, whereas *pgp1-2* elongated the primary root to the wild-type level in the dark (Figure 7f).

### 2.7. Cellular Architecture of the Root Tip Was Disrupted by Loss of Anionic Lipids

**Figure 8:**
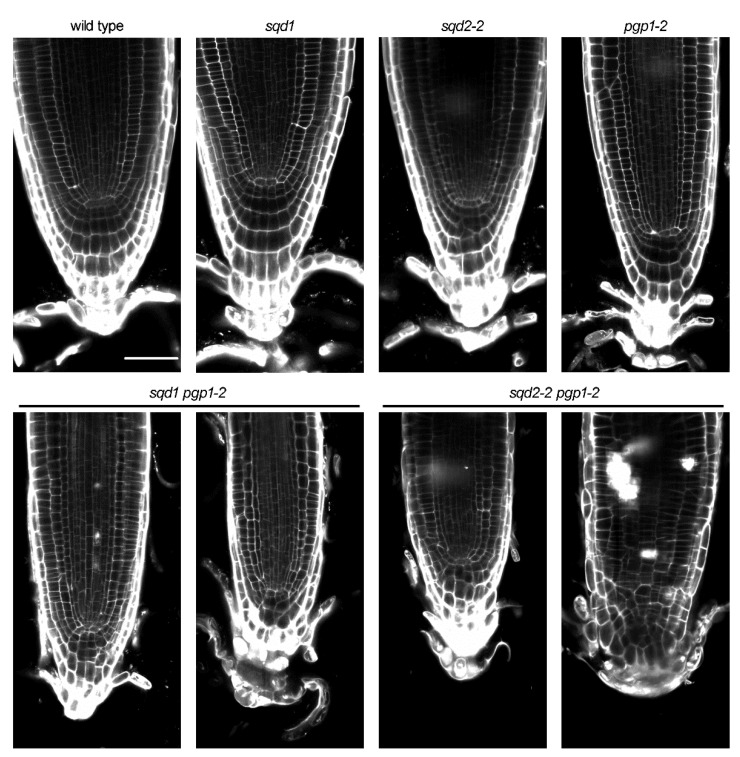
Cellular architecture of root tips of 7-d-old seedlings in wild type and knockout mutants of anionic lipid biosynthesis in plastids. Two different individuals were shown for the *sqd1 pgp1-2* and *sqd2-2 pgp1-2* double mutants. Scale bar = 50 μm.

To examine whether the loss of anionic lipids affects the cellular organization of the root tip, the primary roots of 7-d-old plants were stained with propidium iodide and observed under a confocal fluorescence microscope (Figure 8). No visible difference of cellular architecture at the root tip region was observed among wild type, *sqd1*, and *sqd2-2*. Moreover, the cell organization of the root tip was not notably disrupted in *pgp1-2*, although the primary root elongation was severely decreased in this mutant in the light (Figure 7a–c). By contrast, both the *sqd1 pgp1-2* and *sqd2-2 pgp1-2* double mutants showed irregular cell arrangement at the root cap and the meristematic zone.

### 2.8. Loss of Plastid Anionic Lipids Impairs Embryo Development and Decreases Germination Capacity

**Figure 9:**
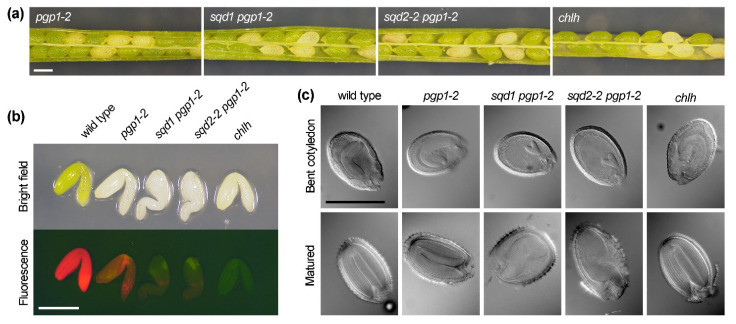
Embryo development in knockout mutants of anionic lipid biosynthesis in plastids. (**a**) Seeds of *pgp1-2*, *sqd1 pgp1-2*, and *sqd2-2 pgp1-2* in the silique at fully developed but before dehydration stages were compared with those of the *chlh* mutant lacking chlorophyll (Chl) biosynthesis. Plants in the heterozygous state for *pgp1-2* or *chlh* were observed. Scale bar = 0.5 mm. (**b**) Embryos were taken out from albino seeds of each mutant line and observed under a fluorescence stereomicroscope along with wild-type embryos. Images of bright field (upper panel) and Chl autofluorescence (lower panel) were shown. Scale bar = 0.5 mm. (**c**) Embryos in albino seeds of each mutant line at the bent cotyledon or matured stage were compared with those of wild type under a differential interference contrast microscope. Scale bar = 0.5 mm.

Because seedling development was severely impaired in the *sqd1 pgp1-2* and *sqd2-2 pgp1-2* double homozygous mutants immediately after germination, we examined whether embryo development was perturbed in these mutants. As reported previously [25], pale yellow seeds, which correspond to the *pgp1-2* homozygote, were detected within the fully developed but still green siliques of the self-pollinated heterozygous *pgp1-2* mutant at a frequency of 25.0% (Figure 9a, Table 2). Similarly, pale yellow seeds were observed in the siliques of the self-pollinated *sqd1*/*sqd1 PGP1*/*pgp1-2* and *sqd2-2*/*sqd2-2 PGP1*/*pgp1-2* plants at a frequency of 24.9% and 24.8%, respectively. To test whether embryo development is arrested in these pale yellow seeds, embryos were taken out from the seeds in fully developed green siliques and observed under a fluorescent stereomicroscope (Figure 9b). Consistent with the previous report [25], the homozygous *pgp1-2* embryos fully developed as did wild type, although they showed an albino phenotype with substantially reduced Chl fluorescence. Embryos from pale yellow seeds in *sqd1*/*sqd1 PGP1/pgp1-2* and *sqd2-2*/*sqd2-2 PGP1/pgp1-2* siliques were also albino and showed only a faint Chl fluorescence. In contrast to the normal morphology of *pgp1-2* embryos, the albino embryos of these double mutants showed a developmental arrest with their hypocotyl irregularly bending. Differential interference contrast microscopy revealed that, unlike in the wild-type and the albino *pgp1-2* seeds, the growth of embryos in the albino seeds of the double mutants was retarded and terminated incompletely (Figure 9c). To assess whether the lack of Chl causes the arrest of embryo development, we observed albino embryos of the *chlh* mutant, which has a knockout mutation in the *CHLH* gene encoding the Mg-chelatase H subunit and completely lacks the activity to synthesize Mg-protoporphyrin IX, an essential intermediate of the Chl biosynthesis pathway [26]. Although the albino *chlh* embryos accumulated no Chl, their morphology was similar to the wild-type embryos (Figure 3b,c), which indicates that Chl accumulation is not essential for embryo development.

Then, we investigated whether the impaired embryo development in the *sqd1 pgp1-2* and *sqd2-2 pgp1-2* double mutants decreases germination capacity of seeds. In wild type, *sqd1* and *sqd2-2*, the percentage of germinated seeds were ≥95% of total seeds sowed on plates. However, the germination of seeds from self-pollinated heterozygous *pgp1-2* decreased to 92%. Moreover, in the seeds from self-pollinated *sqd1*/*sqd1 PGP1/pgp1-2* and *sqd2-2*/*sqd2-2 PGP1/pgp1-2* plants, the germination percentage further decreased to 79% and 84%, respectively. We observed that self-pollinated heterozygous parents produced albino seeds at ~25% frequency (Table 2). However, the emergence of albino seedlings after seed germination was 18.4 % for *pgp1-2*, and it further decreased to 10.2% and 12.0% for *sqd1 pgp1-2* and *sqd2-2*- *pgp1-2*, respectively. From the ~25% frequency of albino seeds in the parent siliques, the germination percentage of homozygous *pgp1-2*, *sqd1 pgp1-2*, and *sqd2-2 pgp1-2* genotypes was estimated as 73.6%, 41.0%, and 48.4%, respectively, which are notably low as compared with wild-type seeds.

## 3. Discussion

This is the first study to characterize the mutants completely lacking both SQDG biosynthesis and PG biosynthesis in plastids. The mutants, *sqd1 pgp1-2* and *sqd2-2 pgp1-2*, showed a developmental arrest during embryogenesis (Figure 9), decreased germinability (Table 2), severe growth impairments, and cell abnormalities in the shoot and root (Figure 4, Figure 7 and Figure 8). Chloroplast development was completely abolished in these mutants (Figure 6) with only faint Chl accumulation (Figure 4) and no PSII activity (Figure 5). All these data demonstrate indispensable roles of plastid anionic lipids in various processes of plant growth. We also compared *sqd1* and *sqd2-2* in the *pgp1-1* background (Figure 1, Figure 2 and Figure 3) in which the PGP1 activity decreases to 30% of the wild-type level [13]. Our analysis allows for distinguishing the role of SQDG and GlcADG and revealed that SQDG, but not GlcADG, is essential for the partial compensation of decreased PG biosynthesis in plastids.

### 3.1. Role of SQDG in Complementing the Function of PG in Chloroplast Development and Photosynthesis

In contrast to the severe impairment of leaf development in the *sqd1 pgp1-2* and *sqd2-2 pgp1-2* double mutants, the relatively milder growth defect of the *sqd1 pgp1-1* and *sqd2-2 pgp1-1* mutants allowed for detailed photosynthetic characterization under P-sufficient and -starved conditions. Under the P-sufficient condition, both *sqd1 pgp1-1* and *sqd2-2 pgp1-1* showed lower Y_II_ levels than *pgp1-1* (Figure 3), which suggests that, in *pgp1-1*, SQDG, but not GlcADG, maintains the photosynthetic electron transport activity to the wild-type levels despite a partial loss of PG. The similar results were observed in the *sqd1* and *sqd2-2* single mutants under the P-starved condition. Although *sqd1* and *sqd2-2* showed wild-type levels of Y_II_ under the P-sufficient condition, these mutants decreased Y_II_ more strongly than wild type and *pgp1-1* with P starvation. In *sqd1* and *sqd2-2*, a decrease in PG by P starvation in addition to the genetic loss of SQDG biosynthesis would cause a crucial lack of anionic lipids in chloroplasts and thereby impair photosynthetic reactions under P starvation. By contrast, in *sqd1 pgp1-1* and *sqd2-2 pgp1-1*, Y_II_ levels were not altered by P starvation because PG content, and thus Y_II_ levels, were low even under the P-sufficient condition. No differences between *sqd1* and *sqd2-2* and between *sqd1 pgp1-1* and *sqd2-2 pgp1-1* under P starvation confirm no contribution of GlcADG to complementing PG functions in photosynthesis. GlcADG would play an important role in cellular processes other than photosynthesis under P starvation.

Although the *pgp1-2* mutant showed much stronger impairments of photosynthetic reactions and chloroplast development than *pgp1-1*, this mutant still contains a small amount of Chl and partially maintained the photosynthetic electron transport activity [15]. However, the additional mutation of either *sqd1* or *sqd2-2* in *pgp1-2* remarkably impaired Chl accumulation and completely abolished the PSII activity (Figure 4 and Figure 5). These data indicate that SQDG functions to maintain the partial Chl accumulation and photosynthetic activity in *pgp1-2*. We previously reported that P starvation, which activated the biosynthesis of glycolipids including SQDG, slightly increased Chl content and thylakoid-like membrane formation in the *pgp1-2* plastids [16]. In this study, P starvation did not increase Chl content in the *sqd1 pgp1-2* and *sqd2-2 pgp1-2* double mutants. Therefore, the increased Chl content in *pgp1-2* under the P-starved condition requires SQDG accumulated in response to P starvation. In contrast to increased Chl content and partial thylakoid development, Fv/Fm in *pgp1-2* did not increase and even decreased under the P-starved condition [16], which may be due to excessive accumulation of Chl over functional PSII with a further decrease in PG content by P starvation. Similarly, in this study, no improvement and, in some *pgp1-2* plants, complete abolishment of Fv/Fm were observed under the P-starved condition (Figure 5). Therefore, SQDG increased during P starvation can partially compensate the loss of PG in terms of Chl accumulation but is unable to maintain the PSII functionality without PG. Essential roles of specific PG molecules in the PSII complex have been revealed in a cyanobacterium *Synechocystis* sp. PCC 6803 [27,28]. The specific functions of PG in PSII may not be replaced by SQDG in plants as well as cyanobacteria. A similar speculation can be made for the PSI complex, which also contains PG molecules as structural components [29,30], although the particular function of each PG molecule in the PSI complex remains unclear. P starvation, which induces GlcADG accumulation in *A. thaliana*, did not positively affect the PSII photochemistry and Chl accumulation in *sqd1 pgp1-2* as compared with *sqd2-2 pgp1-2*, so GlcADG has no capacity to replace the PG molecules in photosynthetic complexes.

Although *sqd1* and *sqd2-2* showed no noticeable difference in the Y_II_ levels compared with wild type under the P-sufficient condition, these mutants showed a slight decrease in Chl content (Figure 1) and increased susceptibility to DCMU (Figure 2). Therefore, although PG is the main anionic lipid for chloroplast development and photosynthesis, SQDG also contributes to these processes along with PG even under P-sufficient conditions. Of note, SQDG molecules are found in PSII of plants and cyanobacteria as structural components [31,32]. Characterization of PSII from a SQDG-deficient mutant of *Thermosynechococcus elongatus* revealed that the loss of SQDG partially impaired the PSII activity by inhibiting electron transfer at the acceptor site [33]. In the SQDG-lacking PSII, other lipids, most likely PG molecules, occupied the SQDG-binding sites. The data in *T. elongatus* suggest that although SQDG molecules in the PSII-binding sites are necessary to fully maintain the activity and stability of PSII, replacement of SQDGs by other lipids, probably PG, can partially complement for their functions, which would lead to only a partial impairment of PSII by lack of SQDG. Because *T. elongatus* and *C. reinhardtii* showed stronger defects in the PSII function and growth by loss of SQDG biosynthesis than *A. thaliana* under P-sufficient conditions [9,21,34], the replaceability of SQDG by PG or other lipids may partially vary with species.

### 3.2. Role of Plastid Anionic Lipids in Root Growth and Embryo Development

The complementary relationship between PG and SQDG has been mainly discussed in relation to their functions in photosynthesis and thylakoid membrane formation. In this study, we revealed that loss of these anionic lipids impaired the primary root growth and embryo development. The *pgp1-1* mutant showed a slight decrease in the primary root growth in the light and the decreased root growth was further enhanced by the additional mutation of *sqd1* or *sqd2-2* with *pgp1-1* (Figure 1). However, these mutants showed no growth impairment of the primary root in the dark, which implies that a light-dependent pathway such as photosynthesis causes the impaired growth of the primary root in these mutants, although the plants were grown in the presence of 1% sucrose. Decreased primary root growth was also reported in the *pgpp1-1* mutant lacking PGP phosphatase 1 (PGPP1), which catalyzes the final step of PG biosynthesis [5]. Because the *pgpp1-1* mutant showed reduced Chl content and slightly impaired chloroplast development [35], decreased photosynthesis may explain in part the decreased root growth. However, in *pgpp1-1*, the impaired root growth was stronger than *pgp1-1*, although the chloroplast dysfunctions were similar to or milder than those in *pgp1-1*. Moreover, a weaker PGPP1 mutant *pgpp1-2* also had a shorter root phenotype, although this mutant had no photosynthetic impairments [5]. Therefore, the disruption of PGPP1 is likely to cause the root growth impairment independently of photosynthesis. Because PGPP1 is targeted to mitochondria in roots [5], PG biosynthesis by PGPP1 in mitochondria may be required for the root growth. As is PGPP1, PGP1 is also targeted to mitochondria in addition to plastids [11]. However, for the PGP biosynthesis, PGP2 localized to ER can complement the role of PGP1 in mitochondria [11,12], which may explain why *pgp1-1* showed a weaker impairment of the primary root growth than *pgpp1-1*. Why disrupted PG biosynthesis, presumably in mitochondria, causes the short root phenotype remains unknown. Because cardiolipin, which is formed from PG in mitochondria, is required for mitochondrial functions and loss of CL impairs root growth in *A. thaliana* mutants [36], PG may be involved in root growth as a substrate of CL biosynthesis. However, we cannot exclude PG-specific functions in root growth, which should be addressed in future studies. The *pgp1-2* mutant also showed a shorter primary root than wild type in the light, but not in the dark (Figure 7). Babiychuk et al. (2003) showed that the *PGP1* knockout mutant accumulated cardiolipin to the wild-type level and developed mitochondria with normal structure and respiratory functions [11]. Therefore, the complete loss of PGP1 would not remarkably affect mitochondrial functions in roots.

In contrast to the *pgp1-2* single mutant, the *sqd1 pgp1-2* and *sqd2-2 pgp1-2* double mutants showed decreased primary root growth even in the dark (Figure 7). Because SQDG is not included in mitochondria at least under P-sufficient conditions [37], the decreased root growth of these double mutants may occur independently of mitochondrial activity. The double mutants showed severe disruption of root tip architecture (Figure 8), which would lead to the shorter primary root of the mutants. Of note, the embryo development is severely retarded in the *sqd1 pgp1-2* and *sqd2-2 pgp1-2* double mutants, but not in the *pgp1-2* single mutant (Figure 9). Therefore, the incomplete plant body development during embryogenesis might be associated with the impaired primary root growth after germination. Photosynthetic dysfunctions would be unrelated to the impaired embryo development in the double mutants because Chl-deficient embryos of *pgp1-2* and *chlh* could fully develop during seed maturation. Abnormal embryo development was reported in several *A. thaliana* mutants related to lipid metabolism in plastids [38,39,40], which includes a knockout mutant for MGDG biosynthesis [41]. Therefore, plastid lipid metabolism, in which anionic lipid biosynthesis is involved, may be crucial for the normal development of embryos. The severe disruption of leaf formation, root tip architecture, and embryo development in *sqd1 pgp1-2* and *sqd2-2 pgp1-2* suggests that anionic lipids synthesized in plastids are essential for cellular organization in the meristematic tissues. Because *pgp1-2* as well as *sqd1* and *sqd2-2* single mutants did not show such severe developmental defects in the meristematic tissues, PG and SQDG can complement each other in these tissues. Considering that PG and SQDG are major glycerolipid constituents in envelope membranes of non-photosynthetic plastids in addition to chloroplasts [42], lack of anionic lipids in the plastid envelope may disrupt plastid functions and/or interorganellar associations required for cellular development and organization. In the dark condition, both *sqd1* and *sqd2-2* mutant showed slightly reduced primary root growth compared with wild type (Figure 1f). Although it might reflect an unknown function of SQDG in the primary root growth in the dark, we cannot make any conclusion because the *sqd1 pgp1-1* and *sqd2-2 pgp1-1* double mutants showed no reduction in the primary root growth in the dark. Again, no differences between *sqd1 pgp1-2* and *sqd2-2 pgp1-2* during embryo and seedling development demonstrate that GlcADG does not have important roles in these processes under P-sufficient conditions. How PG and SQDG are involved in the cellular organization in addition to photosynthetic machinery awaits future investigations.

## 4. Materials and Methods

### 4.1. Plant Materials and Growth Conditions

The wild type, *sqd1* [24], *sqd2-2* [18], *pgp1-1* [13], and *pgp1-2* [14] were the Columbia ecotype of *A. thaliana* (Table 1). The *sqd1 pgp1-1*, *sqd2-2 pgp1-1*, *sqd1 pgp1-2*, and *sqd2-2 pgp1-2* double mutants were generated by crossing each single mutant line. The genotypes for each allele were confirmed by PCR analysis as described [12,13,18,24]. Because the homozygous *pgp1-2* mutation causes a seedling lethal phenotype, the *pgp1-2* allele was maintained in the heterozygous state for the *pgp1-2* single mutant and *sqd1 pgp1-2* and *sqd2-2 pgp1-2* double mutants. In these mutant lines, plants in the homozygous *pgp1-2* state were identified with their albino or pale yellow-green leaf phenotypes in the progeny of self-pollinated parents under the light condition. For etiolated seedlings germinated in the dark, mutants in the homozygous *pgp1-2* state were almost indistinguishable from the heterozygous and wild-type seedlings in their cotyledon color phenotype. Therefore, in the analysis of etiolated seedlings of *pgp1-2*, *sqd1 pgp1-2*, and *sqd2-2 pgp1-2* lines, we first took photographs of all etiolated seedlings for size measurements and then illuminated the seedlings for 2 d to identify albino individuals with homozygous *pgp1-2* allele.

Surface-sterilized seeds on Murashige and Skoog (MS) medium (adjusted to pH 5.7 with KOH) containing 1% (*w/v*) sucrose were cold-treated at 4 °C for 3 days in the dark. Then, plants were germinated and grown in a chamber maintained at 23 °C under continuous white light (~80 μmol photons m^−2^ s^−1^). For the growth phenotype analysis, plants were vertically grown on the MS medium solidified with 0.6% (*w/v*) Gelrite (Wako, Tokyo, Japan). The length of the primary root and the hypocotyl was determined by using ImageJ software (https://imagej.nih.gov/ij/, accessed on 4 May 2021). For the other analysis, plants were horizontally grown on the MS medium solidified with 0.8% (*w/v*) agar. For P-starvation analysis, 7-d-old seedlings of wild type, *pgp1-1*, *sqd1*, and *sqd2-2*, 11-d-old seedlings of *sqd1 pgp1-1* and *sqd2-2 pgp1-1*, and 14-d-old seedlings of *pgp1-2*, *sqd1 pgp1-2*, and *sqd2-2 pgp1-2*, which were roughly at a similar developmental stage with four true leaves, were transferred to the MS medium lacking KH_2_PO_4_ and further grown for 7 d. For DCMU treatment, 7-d-old seedlings were transferred to the MS medium containing several concentrations of DCMU and grown for another 7 d.

To determine germination ability, seeds were collected from adult *A. thaliana* plants grown for ~7 weeks in Giffy 7 pots. After desiccation of collected seeds for a week in a dry container at room temperature, surface-sterilized seeds were incubated on the MS medium in a growth chamber for 4 d, and germination percentage was determined under a stereomicroscope.

### 4.2. Measurement of FW and Chl Content

The shoot of the 14-day-old seedling was cut at the hypocotyl and weighed to determine FW. Then, the shoot was incubated in 80% (*v/v*) acetone at 4 °C in darkness for two days to extract lipophilic pigments. The absorbance of the acetone extract at 720, 663.2, and 646.8 nm was measured with a spectrometer (V-730 BIO, JASCO, Tokyo, Japan) to determine the total content (Chl *a* + Chl *b*) as described [43]. For *pgp1-2*, *sqd1 pgp1-2*, and *sqd2-2 pgp1-2*, the Chl content in a seedling was below a detection limit in the spectrometric analysis. Therefore, for these mutants, intensity of Chl fluorescence at 666 nm under 435 nm excitation was measured with a fluorospectrometer (RF5300, Shimadzu, Kyoto, Japan) and total Chl content was determined by using a known concentration of Chl as a standard.

### 4.3. Analysis of Photosynthetic Efficiency

Chl fluorescence parameters were analyzed by using an imaging PAM fluorometer (IMAGING-PAM MAXI, Walz, Effeltrich, Germany) and ImagingWin software. Samples incubated under dim light (~2 μmol photons m^−2^ s^−1^) for ~30 min were dark-treated for 15 min in the device. After determination of minimal Chl fluorescence (Fo) with the lowest measuring light intensity (~0.2 μmol photons m^−2^ s^−1^ at a frequency of 2 Hz), samples were illuminated with a saturating pulse flash (~3400 μmol photons m^−2^ s^−1^ for 720 ms) to determine maximal Chl fluorescence (Fm). The maximum quantum yield of PSII (Fv/Fm) was calculated as (Fm-Fo)/Fm. For the analysis of light–response curves of effective quantum yield of PSII (Y_II_), plants were illuminated with actinic light of given intensity for 3 min and a second saturating pulse to determine stationary (F) and maximal fluorescence under light (Fm’), respectively, which were used to calculate Y_II_ as (Fm’-F)/Fm’. Photosynthetic photon flux density of actinic light was successively increased every 3 min to 20, 55, 80, 110, 185, 280, 395, 610, and 800 μmol photons m^−2^ s^−1^.

### 4.4. Transmission Electron Microscopic Analysis

The *A. thaliana* leaves were fixed in 4% (*w/v*) glutaraldehyde and 4% (*w/v*) paraformaldehyde, buffered with 50 mM sodium cacodylate at pH 7.0 overnight at 4 °C, and washed with the same buffer at 4 °C for 4 h. Subsequently, they were post-fixed in 2% (*w/v*) OsO_4_ in 50 mM cacodylate buffer at 4 °C for 2 h. The fixed samples were run through an alcohol series and embedded in Spurr’s resin (Polysciences Inc., Warrington, PA, USA). Ultra-thin sections (80 nm thick) were cut with a diamond knife (Diatome; Biel, Switzerland) on an ultra-microtome (Ultracut S; Leica, Vienna, Austria). The floating sections were transferred to formvar-coated grids. They were double stained with 1% (*v/v*) uranyl acetate for 20 min and with lead citrate solution for 10 min. After washing with distilled water, the samples were observed with a JEM-1400 transmission electron microscope (JEOL, Tokyo, Japan).

### 4.5. Root Tip Observation

For the observation of root tip cells, the primary roots of the 7-day-old seedlings were stained with 10 mg ml^−1^ propidium iodide (PI) on microscope slides for ~3 min and observed under a confocal laser scanning microscope (LSM700, Zeiss, Jena, Germany), with double-sided tape used as a spacer between a microscope slide and a coverslip. Root tip cells were visualized with PI fluorescence at 600–640 nm under 555 nm excitation.

### 4.6. Embryo Observation

For the observation of Chl accumulation in embryos, embryos were taken out from seeds that were fully developed but not yet degreened and dehydrated. Chl autofluorescence was observed under a stereomicroscope (MZ 16FA; Leica, Wetzlar, Germany) with a long-pass filter set (TXR, Leica) and a color CCD camera (VB-7010; Keyence, Osaka, Japan) in darkness. For the observation of embryo development in seeds, seeds were collected from siliques at different stages after self-pollination and treated with a clearing solution of chloral hydrate, water, and glycerol (8:2:1, *v/v*/*v*) for 1 h. Then, the embryos in seeds were observed under a differential interference contrast microscope (BX60-34 FL, Olympus, Tokyo, Japan).

## Figures and Tables

**Table 1:** Arabidopsis mutants deficient in plastid anionic lipid biosynthesis used in this study.

Line	Mutation	Lipidic Phenotype	Growth Phenotype	Reference
*sqd1*	T-DNA insertion in the first exon of *SQD1*	Complete lack of SQDG	Wild type-like phenotype under both P-sufficient and -starved conditions	[18]
*sqd2-2*	T-DNA insertion in the first exon of *SQD2*	Complete lack of SQDG and GlcADG	Wild type-like phenotype under a P-sufficient condition and growth defects under a P-starved condition	[17,18]
*pgp1-1*	Single amino acid substitution (P170S) of PGP1	80% reduction of PGP1 activity and 30% reduction of PG content	Slight decrease in chlorophyll content and phtoosynthetic activity	[13]
*sqd1 pgp1-1*	Double mutations of *sqd1* and *pgp1-1*	Complete lack of SQDG and decreased PGP1 activity	Stronger defects in growth, chlorophyll accumulation, and photosynthetic activity than *pgp1-1*	This study (Figure 1, Figure 2 and Figure 3)
*sqd2-2 pgp1-1*	Double mutations of *sqd2-2* and *pgp1-1*	Complete lack of SQDG and GlcADG and decreased PGP1 activity	Almost the same as *sqd1 pgp1-1* in growth and photosynthetic characteristics	This study (Figure 1, Figure 2 and Figure 3)
*pgp1-2*	T-DNA insertion in the first exon of *PGP1* with a start codon deletion	88% reduction of PG content by loss of PG biosynthesis in plastids	Seedling lethal phenotype with severe impairments of chlorophyll accumulation, thyalkoid formation, and photosynthesis	[14,15,16]
*sqd1 pgp1-2*	Double mutations of *sqd1* and *pgp1-2*	Complete lack of SQDG and loss of PG biosynthesis in plastids	Stronger defects in growth, chlorophyll accumulation, thylakoid formation, and photosynthesis than *pgp1-2*	This study (Figure 4, Figure 5, Figure 6, Figure 7, Figure 8 and Figure 9)
*sqd2-2 pgp1-2*	Double mutations of *sqd2-2* and *pgp1-2*	Complete lack of SQDG and GlcADG and loss of PG biosynthesis in plastids	Almost the same as *sqd1 pgp1-2* in growth and photosynthetic characteristics	This study (Figure 4, Figure 5, Figure 6, Figure 7, Figure 8 and Figure 9)

**Table 2:** Percentage of albino seeds, ungerminated seeds, and albino seedlings of wild type and anionic lipid mutants.

Phenotype	Wild Type	*sqd1*	*sqd2-2*	*pgp1-2*	*sqd1 pgp1-2*	*sqd2-2 pgp1-2*
albino seed	0.0%	0.0%	0.0%	25.0%	24.9%	24.8%
	(0/562)	(0/669)	(0/626)	(199/797)	(237/716)	(274/1105)
ungerminated seed	2.9%	5.3%	2.5%	7.9%	21.1%	16.1%
	(8/280)	(17/320)	(7/280)	(110/1370)	(304/1400)	(229/1391)
albino seedling	0.0%	0.0%	0.0%	18.4%	10.2%	12.0%
	(0/280)	(0/320)	(0/280)	(250/1370)	(133/1400)	(166/1391)

Seeds from self-pollinated wild type, homozygous *sqd1*, homozygous *sqd2-2*, heterozygous *pgp1-2* (*PGP1*/*pgp1-2*), and the double mutants in the heterozygous pgp1-2 state (*sqd1*/*sqd1 PGP1*/*pgp1-2* and *sqd2-2*/*sqd2-2 PGP1*/*pgp1-2*) were investigated. Numbers in parentheses indicate the number of albino seeds, ungerminated seeds, or albino seedlings/total in each line.
